# Herpesvirus reactivation in respiratory tract is associated with increased mortality of severe pneumonia patients and their respiratory microbiome dysbiosis

**DOI:** 10.3389/fcimb.2023.1294142

**Published:** 2023-12-22

**Authors:** Yongan Liu, Zhenliang Wen, Yuan Fang, Tao Wang, Fengsheng Wu, Hongming Zhang, Dechang Chen, Jiao Liu

**Affiliations:** ^1^ Department of Critical Care Medicine, Ruijin Hospital, Shanghai Jiaotong University School of Medicine, Shanghai, China; ^2^ Department of Medicine, Genoxor Medical Science and Technology Inc., Zhejiang, China

**Keywords:** herpesvirus reactivation, severe pneumonia, microbiome, mortality, metagenomic next-generation sequencing

## Abstract

Severe pneumonia (SP) is a respiratory tract disease that seriously threatens human health. The herpesvirus detected in patients, especially with severe and immunodeficient diseases, is gradually attracting the attention of clinical doctors. However, little is known about the effect of herpesvirus on the prognosis of SP patients and the pulmonary microbial community. Here, we retrospectively analyzed respiratory samples from 45 SP patients detected by metagenomic next-generation sequencing (mNGS). A total of five types of herpesviruses were detected, with *Human alphaherpesvirus 1* (HHV-1) in 19 patients, *Human betaherpesvirus 5* (CMV) in 7 patients, *Human betaherpesvirus 7* (HHV-7) in 6 patients, *Human alphaherpesvirus 2* (HHV-2) in 5 patients, and *Human gammaherpesvirus 4* (EBV) in 4 patients. Further analysis showed that the mortality of the herpesvirus-positive group was significantly higher than that of the negative group. The results also showed that HHV-1 was significantly associated with the prognosis of SP patients, while the other herpesviruses did not have a significant difference in patient mortality. A comparison of the microbial community characteristics of SP patients showed a significant difference in beta-diversity between herpesvirus-positive and negative groups. Species difference analysis showed that the herpesvirus-positive group was related to more conditional pathogens, such as *Pneumocystis jirovecii* and *Burkholderia cepacia*. In summary, our results suggest that the presence of herpesvirus is associated with the mortality of SP patients. Furthermore, enrichment of conditional pathogens in the respiratory tract of herpesvirus-positive SP patients may be a potential reason for the increased mortality.

## Introduction

Severe pneumonia is a significant public health problem, causing high incidence and mortality worldwide. Despite advancements in medical treatment, severe pneumonia remains a leading cause of mortality in the intensive care unit (ICU) (Jain et al., 2015). Patients with severe pneumonia are at an increased risk of developing secondary infections, including viral infections such as herpesvirus. Herpesvirus are highly contagious pathogens commonly associated with skin and mucosal lesions but can also cause severe respiratory disease in immunocompromised individuals (Sánchez-Ponce et al., 2018). Previous studies have shown that reactivation of herpesvirus in severe pneumonia patients may be associated with prolonged hospital stay, increased ICU admission rates, and increased mortality (Gooskens et al., 2007; Simonnet et al., 2021). However, the potential mechanism that how herpesvirus reactivation impact on the clinical outcomes of severe pneumonia remains unclear.

The respiratory microbiome is a complex ecosystem composed of various microbial communities, including bacteria, viruses, and fungi, that play a crucial role in maintaining respiratory system health by regulating immune response, protecting the host from pathogen invasion, and providing nutrients and metabolites to host cells. The composition and diversity of the respiratory microbiome can be influenced by various factors, including age, smoking history, antibiotic use, and underlying diseases (Faner et al., 2017). Recent studies have shown that changes in the respiratory microbiome are associated with the severity and outcome of pneumonia. Dysbiosis or microbial community imbalance is associated with a prolonged hospital stay, treatment failure, and mortality (Ren et al., 2021). In addition to viral infections, the lower respiratory tract microbiota has been shown to have a significant impact on the development and progression of severe pneumonia. Studies have reported changes in the lower respiratory tract microbiota in patients with severe pneumonia, including the emergence of pathogenic bacteria and a decrease in microbial diversity (Dickson et al., 2020; Woodall et al., 2022). However, there have been few studies on the relationship between the reactivation of herpesvirus in severe pneumonia patients and changes in the lower respiratory microbiome.

In this study, we aimed to investigate the effect of herpesvirus reactivation on the prognosis of severe pneumonia patients and changes in the lower respiratory microbiome. Our study may help to understand better the role of herpesvirus reactivation in the pathogenesis of severe pneumonia.

## Materials and methods

### Clinical specimens

The severe pneumonia patients, diagnosed as CAP/VAP/HAP according to IDSA, included in this study were all from the intensive care unit of Ruijin Hospital in Shanghai. Bronchoalveolar lavage fluid and sputum of these patients were collected from May 2019 to September 2022 for mNGS, and almost all sputum samples are deep respiratory aspirates collected through tracheal intubation. Each sample was sent to the laboratory immediately after collection and detected within 24 hours after collection. All sample information is presented in **Table S1**. This study has obtained ethical approval from the local medical ethics committee at Ruijin Hospital, and each enrolled patient or their family signed a written informed consent. Other detection data corresponding to the time when performed mNGS (within two days) were collected, including infective and immune testing, ICU length of stay, sequential organ failure assessment (SOFA), Acute Physiology and Chronic Health Evaluation (APACHE II) scores, underlying disease, and findings on other routine examinations.

### DNA extraction and shotgun sequencing

Sputum specimens were resuspended in approximately 200 mL of sputum with dithiothreitol (DTT) and proteinase K solution, and incubated at 56°C for 20 minutes. After centrifugation, host background nucleic acids and cell debris were removed. Total DNA was extracted using HostZERO Microbial DNA Kits (Zymo Research, USA) according to the manufacturer’s instructions, and DNA yield was quantified using the Quant-iT dsDNA HS assay kit and Qubit 3.0 fluorometer (Thermo Fisher Scientific, USA). DNA fragments were enzymatically sheared to an appropriate length of approximately 200 bp. Subsequently, a Nextera XT DNA library preparation kit (Illumina, USA) was used to construct libraries, and 1 µg of the metagenomic library was obtained by 5-7 cycles of PCR. The quality of the library was assessed using high-sensitivity DNA analysis on a 2100 Bioanalyzer (Agilent Technologies, USA). Shotgun metagenomic sequencing was performed on Illumina NextSeq 550 using the NextSeq High Output kit in SE75 strategy.

### Bioinformatics analysis

A series of processing steps were applied to the raw sequencing reads to obtain quality-filtered reads for further analysis and reduce duplication. First, Cutadapt (v1.2.1) was used to remove sequencing adapters. Then, a sliding-window algorithm in fastp was employed to trim low-quality reads. Next, BMTagger was used to align reads to the human reference genome GRCh37 and remove any host contamination. After quality-filtered reads were obtained, Kraken2 was used to perform taxonomical classifications of metagenomics sequencing reads from each sample against a custom k-mer database, which included genomes from more than 27,000 species of archaea, bacteria, viruses, fungi, protozoans, and metazoans sourced from the NCBI assembly databases. Reads assigned to metazoans were subsequently discarded for downstream analysis.

### Statistical analyses

The study recorded the survival of severe pneumonia patients within 90 days and plotted a survival curve using GraphPad Prism 9. The log-rank (Mantel-Cox) test was used to compare the survival curves. The alpha diversity index was utilized to evaluate the classification diversity of each sample, and the KW test was employed to compute differences between the two groups. Compositional variation of microbial communities across samples was investigated through beta diversity analysis using the Weighted UniFrac distance metrics and was visualized with nonmetric multidimensional scaling (NMDS). The LEfSe (Linear discriminant analysis effect size) was used to detect differentially abundant taxa across groups based on the non-redundant gene taxonomic profiles, using the default parameters. We also conducted ANCOM-BC for differential abundant taxa analysis. The R language package was downloaded from the web: https://github.com/FrederickHuangLin/ANCOMBC. Parameter HOIM was used for P-value correction.

## Results

### Clinical characteristics of patients in this study

**Figure 1:**
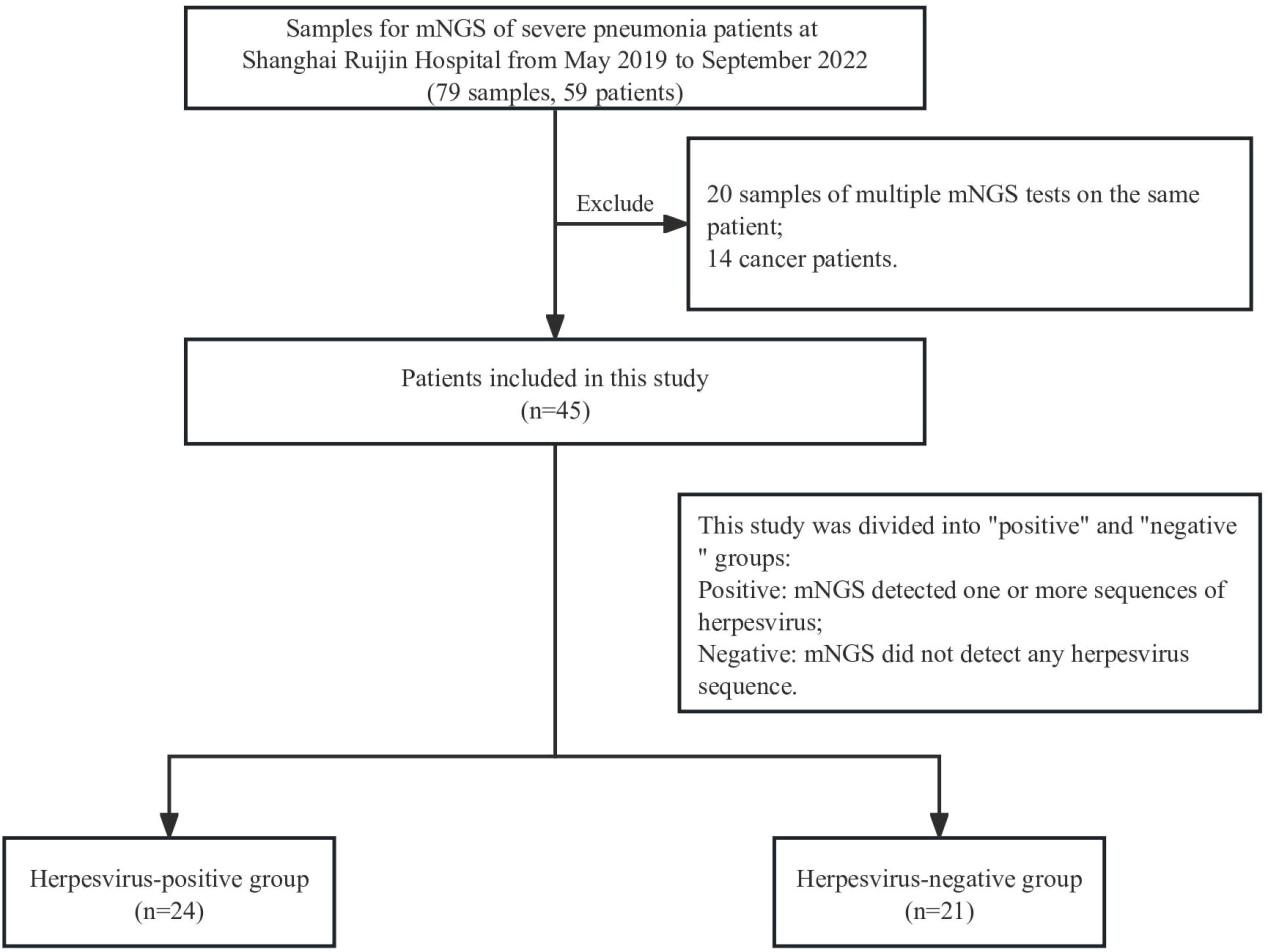
Flow chart of the study design.

In this retrospective study, 59 severe pneumonia patients were admitted in the intensive care unit of our hospital from May 2019 to September 2022, and 79 respiratory samples collected from them were tested by mNGS. Samples from the same patient that underwent multiple mNGS tests were excluded, except for the first one from these patients. Analysis of HSV positive rate and underlying diseases found a significant positive correlation between cancer and HSV (12/14 herpesvirus positive, p=0.0065). All 14 cancer patients were therefore excluded in order to avoid the impact of underlying diseases on the results. Finally, 45 cases of severe pneumonia were included in the final analysis, and each patient matched one sample. The patients were divided into positive or negative groups according to the presence of herpesvirus in mNGS results (**Figure 1**). Demographic and clinical characteristics of patients are shown in **Table 1**. The median age of the positive group was 69.5 years (interquartile range: 46-90) with 18 males (75%); the median age of the negative group was 72 years (interquartile range: 51-85) with 15 males (71.4%). Diabetes (15/45, 33.33%), respiratory system diseases (7/45, 9.3%), and renal system diseases (9/45, 20%) were the most common underlying diseases among patients. Laboratory results showed that the median values of interleukin-2 (IL-2) and procalcitonin (PCT) in the positive group were 656 (interquartile range: 4-795) and 2.215 (interquartile range: 0.3925-13.32), respectively, which were significantly higher than those in the negative group (p=0.037 and 0.045, respectively). There was also a significant difference in mechanical ventilation duration between the two groups (p=0.041), with a higher duration in positive group. The median APACHE II score of the positive group was 17.5 (interquartile range:12-27.75), and the median SOFA score was 6.5 (interquartile range: 3.5-11.75); the median APACHE II score of the negative group was 16.5 (interquartile range: 12.5-19.75) and the median SOFA score was 7 (interquartile range: 5-10.75).

**Table 1:** Demographic and Clinical Characteristics of 45 Cases of Severe Pneumonia. p < 0.05 is considered statistically significant and shown bold.

Characteristics	Positive (n=24)	Negative (n=21)	P value
Age, year (range)	69.5(46-90)	72(51-85)	0.715
Gender (n)			
Male	18	15	
Female	6	6	
Primary diseases (n)			
Diabetes	8	7	
Respiratory Diseases	4	3	
Cardiovascular Disease	3	3	
Renal system Diseases	2	7	
Cerebrovascular Diseases	2	4	
Laboratory tests			
WBC, 10^9/L	11.27(5.995-17.425)	9.61(6.89-12.8)	0.554
NE, 10^9/L	10.53(5.04-14.335)	8.565(6.2825-10.79)	0.735
NE, %	91(82.6-93.9)	86.65(76.2-90.05)	0.072
LYM, 10^9/L	0.66(0.485-0.87)	0.86(0.56-1.175)	0.083
LYM, %	5(3.8-9.2)	8.3(5.775-12.25)	0.128
IL-1, pg/ml	5(5-6.6)	5(2.7-9.75)	0.707
IL-2, pg/ml	656(3.9-795)	3.15(2.4-434.35)	**0.037**
IL-10, pg/ml	5.05(5-12.8)	5(2.85-18.75)	0.337
IL-8, pg/ml	27.6(11.1-70.5)	29.8(2.6-63)	0.873
IL-6, pg/ml	18.5(5.71-363.8)	173.1(13-731.3)	0.468
CRP, mg/L	107(68.25-196.5)	90(16.5-155)	0.417
PCT, µg/L	2.215(0.3925-13.32)	0.41(0.19-1.665)	**0.045**
CD3, cell/mm^3^	598.5(253.5-1071.75)	192(65-492.5)	0.059
CD4, cell/mm^3^	445(141-727.75)	110(28-323)	0.059
CD8, cell/mm^3^	146.5(99.75-309.5)	71(26-189)	0.289
LDH, U/L	401.5(231.75-680.75)	278(215.5-373.5)	0.293
Severity of illness			
Ventilation time(days)	13.5(9-29)	7.5(3.25-15.75)	**0.041**
APACHE II score before treatment	17.5(12-27.75)	16.5(12.5-19.75)	0.645
SOFA score before treatment	6.5(3.5-11.75)	7(5-10.75)	0.803
PaO2/FiO2, mmHg	267.5(203.5-356.75)	277(207-332.25)	0.906
ICU outcomes(days)			
Length of stay in ICU	20(12.25-35.75)	16(12.5-24)	0.322

Patient physiological index measurements are presented as median (interquartile). p < 0.05 is considered statistically significant and shown bold. WBC, white blood cell; NE, neutrophilic granulocyte; LYM, Lymphocyte Count; CRP, C-Reactive Protein; PCT, procalcitonin; APACHE, acute physiology and chronic health evaluation; SOFA, sequential organ failure assessment.

### The pathogen spectrum of severe pneumonia

**Figure 2:**
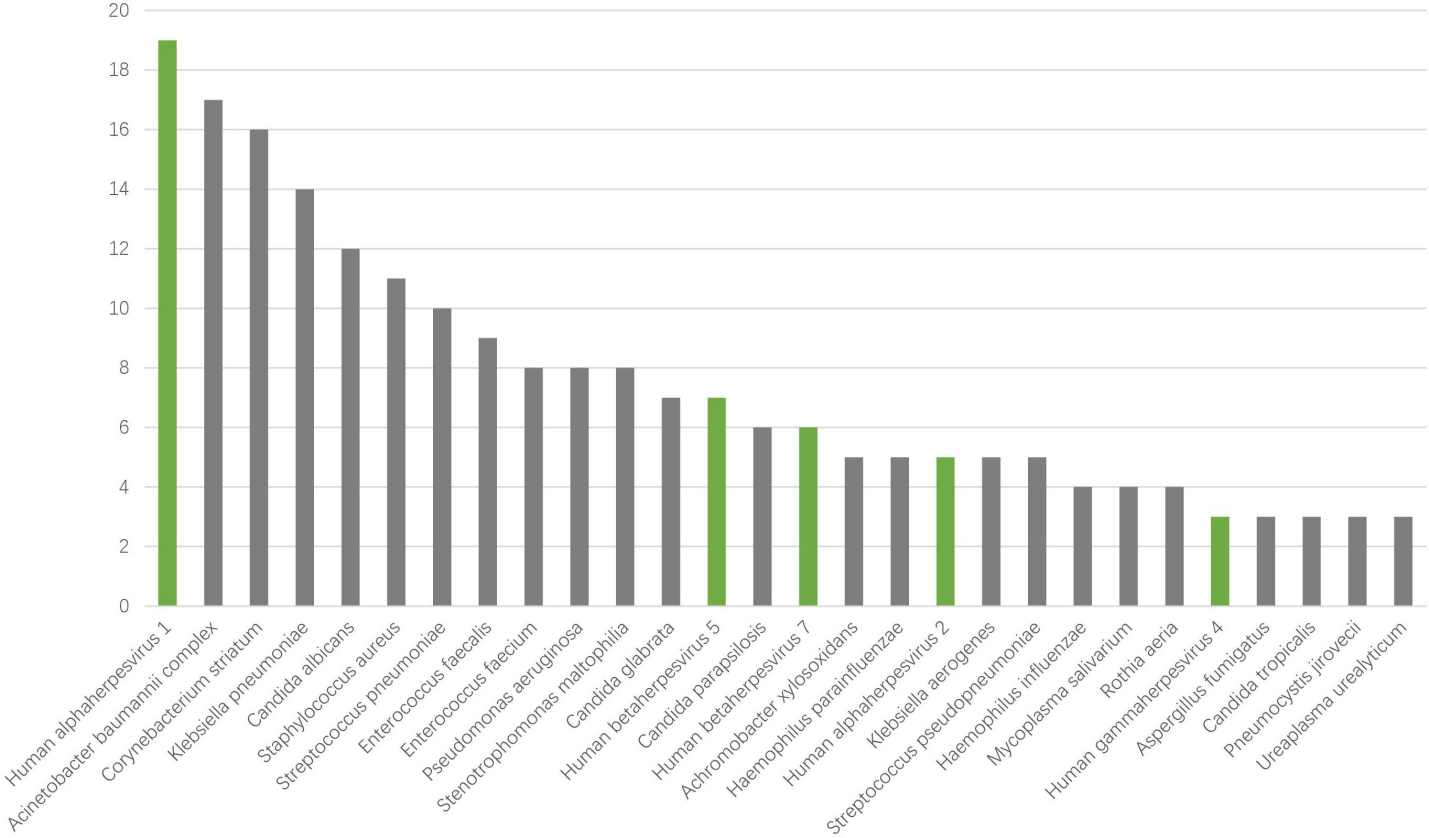
Distribution of bacteria, fungi, and viruses detected by mNGS.

To identify the possible pathogens in severe pneumonia patients, we used mNGS to detect respiratory samples and created a pathogen spectrum. There were 77 types of pathogens were detected in the 45 samples, including bacteria, fungi, viruses and mycoplasma, with 56, 15, 6 and 1 species respectively (**Figure 2**). The top 5 species belonging to bacteria were *Acinetobacter baumannii complex*, *Corynebacterium striatum*, *Klebsiella pneumoniae*, *Staphylococcus aureus*, *Streptococcus pneumoniae*. The top fungi were mainly *Candida* genus, like *Candida albicans*, *Candida glabrata*, *Candida parapsilosis*. The 6 viruses were *Human alphaherpesvirus 1* (HHV-1), *Human alphaherpesvirus 2* (HHV-2), *Human gammaherpesvirus 4* (EBV), *Human betaherpesvirus* 5 (CMV), *Human betaherpesvirus 7* (HHV-7) and *Primate erythroparvovirus 1* (B19). In particular, HHV-1 was the most common species in all kinds of pathogens. The results suggest that herpesviruses may be of great significance for severe pneumonia patients.

### The association between herpesvirus and the mortality of severe pneumonia patients

**Figure 3:**
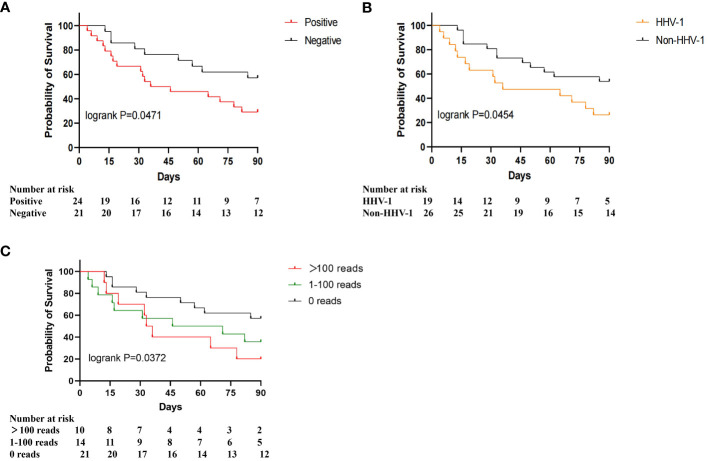
90-day survival proportions for different herpesviruses. **(A)** Survival proportions for herpesvirus-positive and herpesvirus-negative patients; **(B)** Survival proportions for HHV-1 positive and HHV-1 negative patients; **(C)** Survival proportions for >100 reads, 1-100 reads, 0 reads.

To study the association between herpesviruses and the prognosis of patients with severe pneumonia, we plotted the 90-day survival curves for the herpesvirus-positive and herpesvirus-negative groups. As shown in **Figure 3A**, the 90-day mortality was significantly higher in the positive group than in the negative group (70.83% vs. 42.86%, p=0.0471). According to the results of mNGS detection, at least one kind of herpesvirus was detected in 24 patients (53.33%, 24/45), including 19 cases of *Human alphaherpesvirus 1* (HHV-1), 5 cases of *Human alphaherpesvirus 2* (HHV-2), 3 cases of *Human gammaherpesvirus 4* (EBV), 7 cases of *Human betaherpesvirus 5* (CMV), and 6 cases of Human betaherpesvirus 7 (HHV-7). Further investigation of the association between different herpesviruses and the mortality was conducted. The results indicated that the prognosis of the HHV-1-positive group was significantly worse than that of the HHV-1-negative group (73.68% vs. 46.15%, p=0.0454), as shown in **Figure 3B**. The survival curve of the HHV-2-positive, EBV-positive, and HHV-7-positive groups were similar to those of the corresponding negative groups. To investigate the relationship between viral loads of herpesvirus and prognosis, we divided patients into three groups based on the max read counts among all herpesviruses in each sample: >100 reads, 1-100 reads, 0 reads. The survival curve results showed a significant negative correlation between virus load and 90-day survival, as shown in **Figure 3C**. These results suggest that the presence of herpesviruses, especially HHV-1, in respiratory specimens is associated with increased mortality in severe pneumonia patients.

### The association between herpesvirus and the microbial community of sputum in severe pneumonia patients

**Figure 4:**
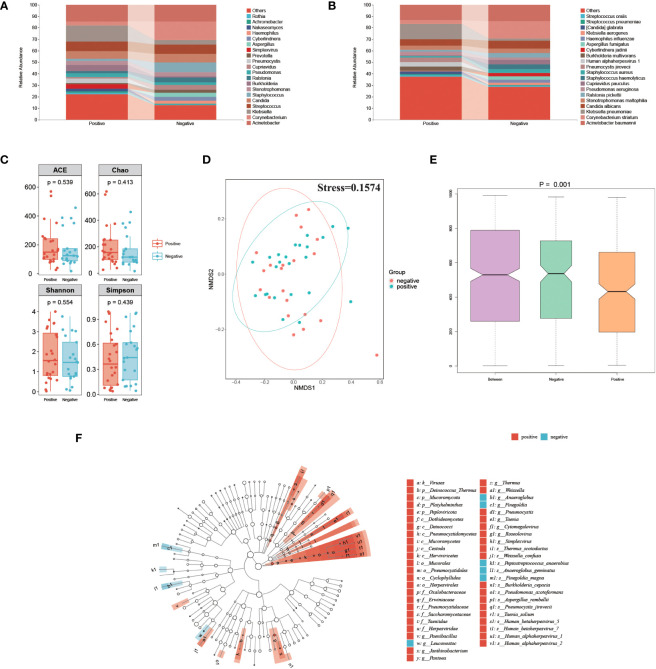
Comparison of respiratory microbiota between herpesvirus-positive and herpesvirus-negative groups. **(A)** Genus-level species composition chart. **(B)** Species-level species composition chart. **(C)** Alpha diversity of each group. **(D)** NMDS plot. **(E)** ANOSIM boxplot. **(F)** LEfSe analysis showed significant differences in abundant biological marker genus between the herpesvirus-positive and herpesvirus-negative groups.

To explore the association between the herpesvirus and the respiratory microbiota of SP patients, we compared and analyzed the metagenomic data between positive and negative groups. The percentage abundance distribution of microorganisms is shown in **Figures 4A, B**. At the genus level, *Acinetobacter*, *Corynebacterium*, *Klebsiella*, *Streptococcus*, *Candida*, and *Staphylococcus* dominate the microbiota in both groups. Compared to the herpesvirus-negative group, patients in the herpesvirus-positive group had lower abundances of *Corynebacterium* and *Staphylococcus* but higher abundance of *Klebsiella*. At the species level, *Acinetobacter baumannii*, *Corynebacterium striatum*, and *Klebsiella pneumoniae* were dominant in both groups, but *Klebsiella pneumoniae* was significantly higher in the herpesvirus-positive group. Alpha diversity analysis showed no significant differences in richness and diversity between the herpesvirus-positive and herpesvirus-negative groups (**Figure 4C**). NMDS analysis of Weighted UniFrac distance (**Figure 4D**) showed that the microbiota in the herpesvirus-positive group was not apart from that in the herpesvirus-negative group (stress=0.1574). However according to the ANOSIM test, there was significant differences in the microbiota structure between the two groups (**Figure 4E**, p= 0.001). These results suggest that there may be differences in respiratory microbiota between the two groups. We performed LEfSe analysis to determine specific taxonomic groups with abundance changes. The significantly-different species (excluding herpesvirus) between the two groups were shown in **Figure 4F**. *Weissella confusa*, *Pneumocystis jirovecii*, *Pseudomonas azotoformans*, and *Burkholderia cepacia* (potential opportunistic pathogens) were significantly enriched in the herpesvirus-positive group. In contrast, *Finegoldia magna* and *Anaeroglobus geminatus* (potential opportunistic pathogens) were significantly enriched in the herpesvirus-negative group. ANCOM-BC analysis showed the same species in herpesvirus-positive group. However, *Finegoldia magna* and *Anaeroglobus geminatus* were marked false positive, even though p < 0.01(correspondingly 0.004 and 0.009). These results indicate that more opportunistic pathogens are enriched in the respiratory tract of herpesviruses-positive patients with severe pneumonia.

## Discussion

There is a close relationship between critically ill patients with respiratory diseases and herpesviruses. Study showed that the high mortality in patients with Acute Respiratory Distress Syndrome (ARDS) is significantly associated with lower immune phenotypes and lower respiratory tract herpesvirus infections (Bonizzoli et al., 2016). Additionally, the viral load of herpesviruses in bronchoalveolar lavage fluid of severely ill patients is related to their poor prognosis (Linssen et al., 2008; Bonizzoli et al., 2016; Blomberg et al., 2019; Jellinge et al., 2021; Xu et al., 2023). In this study, we conducted mNGS on respiratory samples to analyze the pathogen spectrum and the impact of herpesviruses on prognosis and the respiratory microbiome. Results also showed that the presence of herpesviruses was associated with high mortality in severe pneumonia patients, as well as the changes in respiratory microbiome.

Our results showed that the 90-day mortality was significantly higher in the herpesvirus-positive group compared to the negative group. These findings are consistent with previous studies on severe pneumonia patients, which identified herpesvirus infection as an independent risk factor for predicting poor prognosis (Bonizzoli et al., 2016; Blomberg et al., 2019; Jellinge et al., 2021; Xu et al., 2023). Another meta-analysis conducted by Stefan Hagel et al. suggested that antiviral therapy may reduce the 30-day mortality in mechanically ventilated ICU patients with positive respiratory HSV, while the authors point out that this result must be interpreted with great caution due to the high risk of bias and limited number of patients (Hagel et al., 2020). Our results indicate that the increased mortality may be mainly due to the significant impact of HHV-1. In previous studies, HHV-1 has been proven to be associated with increased mortality in severe COVID-19 patients (Meyer et al., 2021; Giacobbe et al., 2022; Pérez-Pedrero Sánchez-Belmonte et al., 2023). These may explain why antiviral therapy according to HHV1 may benefit for mechanically ventilated patients (Heimes et al., 2020). The relationship between HSV and increased mortality has been widely discussed, still more research is needed to elucidate the clinical benefits and strategies of antiviral therapy in patients with severe pneumonia.

There is an overlap between the bacterial populations observed in the lower respiratory tract and the oropharynx, and under mechanical ventilation, ecological imbalance occurs as microbial diversity diminishes. The interaction between lung microbiota and the host, as well as ecological imbalance, may play a key role in the pathophysiology of chronic inflammatory diseases such as asthma, chronic obstructive pulmonary disease, and cystic fibrosis (Fromentin et al., 2021). Zhao et al. used mNGS data from respiratory samples to screen biomarkers and establish a model for predicting the prognosis of severe pneumonia patients. Unfortunately, no herpesvirus is included as a biomarker (Zhao et al., 2023). Our results align with another study that found an association between herpesvirus reactivation and lung microbiota (Pan et al., 2022). On the other hand, the presence of herpesviruses may alter the respiratory environment, leading to changes in the structure of the microbiome. The loss of microbial diversity in the lower respiratory tract can lead to an imbalance in the community, potentially impairing immune responses, increasing susceptibility to viral infections, and promoting the growth of conditional pathogens. Along with changes in biochemical parameters, this may result in severe forms of diseases (Hernández-Terán et al., 2023).

The presence of herpesviruses may provide opportunities for the growth of these potential pathogens, thereby increasing the risk of infection in severe pneumonia patients. To validate the hypothesis, we further identified specific taxonomic groups with significant abundance differences between herpesvirus-positive and negative groups, including *Weissella confusa*, *Pneumocystis jirovecii*, *Pseudomonas azotoformans*, and *Burkholderia cepacia*, which exhibited significantly higher abundance in the herpesvirus-positive group. These microbes may play a role in exacerbating disease risk for pneumonia patients or those with compromised immune systems (Ganesh et al., 2020; Sivaraj et al., 2020; Takahashi et al., 2022). Multiple microbial infections are often associated with increased severity of disease and poorer prognosis. Various infecting microorganisms can synergistically interact to induce virulence characteristics, alter the infection niche, or modulate host immune responses, promoting microbial infection and contributing to premature patient death (Tay et al., 2016). Another hypothesis is that the reactivation of herpesvirus could primarily only reflect the local immunosuppressive state, but not be pathogenic. In this situation, the role of HSV is similar to that of Torque Teno virus (TTV). TTV is considered a non-pathogenic virus, while an immune marker (Gore et al., 2023). The enrichment of conditional pathogens may be related to the immunosuppressive state. When the viral load gradually increases and the species has a certain pathogenicity, viral infection may occur and the clinical outcomes of patients may be worse. TTV and HSV are viruses commonly discussed in sepsis and organ transplantation, as patients are usually immunosuppressive. Pan et al. investigated the relationship between viruses and prognosis in lung infections in patients undergoing solid organ transplantation. The results showed that CMV was associated with poor prognosis, while TTV was not (Pan et al., 2022).

However, there are limitations in this study. Firstly, our study is retrospective, so causal relationships cannot be drawn. Further prospective studies would help to confirm the association between herpesviruses and mortality, as well as their impact on respiratory microbiota. Secondly, we only analyzed the relationship between the presence of herpesviruses and patient prognosis and the structure of the microbiome. Although we have tried our best to eliminate the influencing factors, other factors such as bacteria genus *Klebsiella* (pathogenic bacteria) may also have an impact on the results. Lastly, due to the limited sample size, larger-scale studies may be needed to provide a more comprehensive evaluation of the relationship between herpesviruses and mortality of severe pneumonia.

## Conclusion

In this study, we performed pathogen testing using mNGS technology on respiratory samples from severe pneumonia patients and analyzed the association between herpesviruses and patient prognosis, as well as the respiratory microbiome. The results showed diverse pathogens in severe pneumonia patients, and the reactivation of herpesviruses is associated with mortality, particularly with the presence of HHV-1. Additionally, the reactivation of herpesviruses may be related to the dysbiosis of the respiratory microbiome in severe pneumonia patients, leading to an enrichment of opportunistic pathogens in the respiratory tract. These findings indicate that clinical practitioners should focus on herpesviruses when managing severe pneumonia patients to improve patient prognosis.

## Data availability statement

The sequencing raw data for this study is public and can be found in NCBI database (PRJNA1017160, mNGS data of severe pneumonia).

## Ethics statement

This research study was conducted retrospectively from data obtained for clinical purposes. Our study passed the review of the Medical Ethics Committee of Ruijin Hospital. Written informed consent was obtained from the patients or their families. All the authors listed have approved the manuscript that is enclosed.

## Author contributions

YL: Data curation, Funding acquisition, Project administration, Writing – original draft, Writing – review & editing. ZW: Data curation, Funding acquisition, Writing – review & editing. YF: Conceptualization, Methodology, Writing – review & editing, Supervision. TW: Data curation, Funding acquisition, Writing – review & editing. FW: Conceptualization, Data curation, Methodology, Project administration, Writing – original draft, Writing – review & editing. HZ: Project administration, Writing – original draft, Methodology. DC: Conceptualization, Funding acquisition, Writing – review & editing. JL: Conceptualization, Funding acquisition, Writing – review & editing, Supervision.
